# Army Medical Intelligence—Official

**Published:** 1864-10

**Authors:** 


### ARMY MEDICAL INTELLIGENCE—OFFICIAL.

### PRINCIPAL HOSPITALS IN THE CONFEDERATE STATES

[CONCLUDED]

#### SOUTH CAROLINA.

| Hospital. | Location. | Surgeon in Charge. |
|---|---|---|
| Ladies' Gen'l No. 3 | Columbia | R. H. Edmonds. |
| General, No. 1 | " | W. C. Horlbeck. |
| Second N. Carolina | " | A. W. Thomson. |
| Way | Kingsville | J. A. Pleasants. |
| Third N. Carolina | Charleston | T. B. Memminger. |
| First S. Carolina | " | G. R. C. Todd. |
| First Louisiana | " | R. Lebby. |
| General | Georgetown | B. C. Fishburne. |
| First Georgia | Charleston | N. H. Cumming. |
| Soldiers' Relief | " | W. H. Huger. |
| Way | Florence | T. A. Dargan. |
| " | Greenville | G. S. Trezevant. |

#### GEORGIA.

| Hospital. | Location. | Surgeon in Charge. |
|---|---|---|
| Erwin | Barnesville | J. A. Groves. |
| Way | Fort Gaines | E. W. McCreery. |
| General, No. 1 | Savannah | W. G. Bulloch. |
| " " 2 | " | W. R. Waring. |
| " | Columbus | J. S. White. |
| Third Georgia | Augusta | J. F. McGeddings. |
| Hardee | Forsyth | Wm. Webb. |
| Clayton | " | Jno. Patterson. |
| General | Guyton | W. S. Lawton. |
| Lumpkin | Covington | E. McDonald. |
| Asylum | Madison | H. H. Clayton. |
| Kingston | Kingston | G. W. McDade. |
| Polk | Atlanta | R. Battey. |
| Bragg | Newnan | J. Gore. |
| Foard | " | J. N. Hughes. |
| Buckner | " | W. T. McAllister. |
| Cannon | La Grange | L. W. Tuttle. |
| St. Mary's | " | J. M. Henson. |
| Law | " | A. Erskine. |
| Oliver | " | J. Williams. |
| Hood | Covington | D. H. Morrison. |
| Dawson | Greensboro' | J. D. Smith. |
| Gilmer | Marietta | P. H. Otey. |
| Academy | " | F. Hawthorne. |
| Foard | " | J. B. Barnett. |
| Bell | Greensboro' | H. V. Miller. |
| Blackie | Madison | J. T. M. Law. |
| Prin | Griffin | L. C. Pyncham. |
| Director | " | R. M. Lyttle. |
| Quintard | " | Asst. Surg. S. V. D. Hill |
| Stout | Madison | J. W. Glenn. |
| Newsom | Thomaston | A. Hunter. |
| Fair Ground, No. 1 | *near* Macon | G. G. Crawford. |
| " " " 2 | " | H. W. Brown. |
| Empire | " | W. P. Harden. |
| Grant | " | J. C. Mullens. |
| Institute | " | D. C. O'Keefe. |
| Hill | Covington | W. H. Robertson. |
| Ocmulgee | Macon | S. E. Chaillè. |
| City Hall | " | L. L. Saunders. |
| Blind School | " | Geo. F. Cooper. |
| Floyd House | " | E. J. Roach. |
| Catoosa | Griffin | C. L. Herbert. |
| Medical College | *near* Macon | W. F. Westmoreland. |
| First Florida | Fort Gaines | J. F. M. Gaston. |
| Reid | West Point | J. W. Oslin. |
| Gamble | Newnan | K. C. Divine. |
| Marshall | Columbus | T. A. Means. |
| Stout | Macon | J. Parker. |
| Lee | Columbus | W. A. Robertson. |

#### ALABAMA.

| Hospital. | Location. | Surgeon in Charge. |
|---|---|---|
| Nott | Mobile | G. A. Nott. |
| General | " | W. C. Cavanaugh. |
| " | Greenville | Asst. Surg. R. B. Maury. |
| Way | Demopolis | H. Hinckley. |
| General | Tuscaloosa | R. N. Anderson. |
| " | Selma | A. Hurt. |
| Way | Talladega | G. S. Bryant. |
| General | Spring Hill | G. Owen. |
| " (Ross) | Mobile | S. L. Nidelet. |
| Way | Selma | W. Curry. |
| Ladies | Montgomery | T. F. Duncan. |
| Stonewall | " | W. M. Cole. |
| Way | Eufaula | P. D. L. Baker. |
| General, (Canty) | Mobile | W. Henderson. |
| " (Levart) | " | R. H. Redwood. |
| Madison House | Montgomery | C. J. Clark. |
| Texas | Auburn | Asst. Surg. L. A. Bryan. |
| St. Mary's | Montgomery | J. H. Watters. |
| General | Notasulga | U. R. Jones. |
| Concert Hall | Montgomery | W. J. Holt. |
| Watts | " | F. M. Hereford. |
| Officers' | Uniontown | G. C. Gray. |
| General | Shelby Springs | B. H. Thomas. |

#### MISSISSIPPI.

| Hospital. | Location. | Surgeon in Charge. |
|---|---|---|
| General | Grenada | J. L. Thompson. |
| Way | Guntown | J. M. Hoyle. |
| | Liberty | R. L. Luckett. |

#### FLORIDA.

| Hospital. | Location. | Surgeon in Charge. |
|---|---|---|
| General | Quincy | J. H. Thompson. |
| " | Tallahassee | E. Geddings. |
| " | Lake City | J. S. Morel. |
| Way | Madison | Asst. Surg. J. Cohen. |

#### TENNESSEE.

| Hospital. | Location. | Surgeon in Charge. |
|---|---|---|
| General | Bristol | R. D. Hamilton. |
| Hood | " | Asst. Surg. J. T. Love. |

### MEDICAL LABORATORIES.

Surgeon A. S. Piggott.....In charge at Lincolnton, North Carolina.

" R. M. Johnson .. In charge at Tyler, Texas.

" W. H. Prioleau...In charge at Macon, Georgia.

" G. L. Blackie.....In charge at Augusta, Georgia.

" J. J. Chisolm.....In charge at Columbia, S. C.

